# Quantification of intracellular payload release from polymersome nanoparticles

**DOI:** 10.1038/srep29460

**Published:** 2016-07-11

**Authors:** Edoardo Scarpa, Joanne L. Bailey, Agnieszka A. Janeczek, Patrick S. Stumpf, Alexander H. Johnston, Richard O. C. Oreffo, Yin L. Woo, Ying C. Cheong, Nicholas D. Evans, Tracey A. Newman

**Affiliations:** 1 Centre for Human Development, Stem Cells and Regeneration, University of Southampton Faculty of Medicine, Tremona Road, Southampton, SO16 6YD, United Kingdom; 2 Institute for Life Sciences, Centre for Biological Sciences, B85, University Road, University of Southampton, United Kingdom; 3 Department of Obstetrics and Gynaecology, Faculty of Medicine, University of Malaya, Kuala Lumpur, 50603, Malaysia; 4 University of Malaya Cancer Research Institute (UMCRI), University of Malaya, Kuala Lumpur, 50603, Malaysia; 5 Bioengineering Sciences Group, Faculty of Engineering and the Environment, University of Southampton, Highfield, Southampton, SO17 1BJ, United Kingdom; 6 Clinical and Experimental Sciences, Medicine, University of Southampton, SO17 1BJ, United Kingdom

## Abstract

Polymersome nanoparticles (PMs) are attractive candidates for spatio-temporal controlled delivery of therapeutic agents. Although many studies have addressed cellular uptake of solid nanoparticles, there is very little data available on intracellular release of molecules encapsulated in membranous carriers, such as polymersomes. Here, we addressed this by developing a quantitative assay based on the hydrophilic dye, fluorescein. Fluorescein was encapsulated stably in PMs of mean diameter 85 nm, with minimal leakage after sustained dialysis. No fluorescence was detectable from fluorescein PMs, indicating quenching. Following incubation of L929 cells with fluorescein PMs, there was a gradual increase in intracellular fluorescence, indicating PM disruption and cytosolic release of fluorescein. By combining absorbance measurements with flow cytometry, we quantified the real-time intracellular release of a fluorescein at a single-cell resolution. We found that 173 ± 38 polymersomes released their payload *per* cell, with significant heterogeneity in uptake, despite controlled synchronisation of cell cycle. This novel method for quantification of the release of compounds from nanoparticles provides fundamental information on cellular uptake of nanoparticle-encapsulated compounds. It also illustrates the stochastic nature of population distribution in homogeneous cell populations, a factor that must be taken into account in clinical use of this technology.

Polymersomes (PMs) are nano-sized artificial vesicles made from synthetic polymers such as poly(ε-caprolactone)-*block*-poly(ethylene glycol), (PEG-*b*-PCL). PMs formed from amphiphilic PEG-*b*-PCL block copolymers self-assemble in aqueous solution with the hydrophobic PCL units forming a spherical membrane surrounded by a hydrophilic PEG corona. PMs can be used to carry and deliver a variety of payloads including plasmids^1^, low molecular weight compounds^2^ and proteins^3^ into cells. Furthermore, PMs can be readily modified to display surface moieties, e.g. short peptide sequences that promote uptake or confer specificity to certain cell types^4^. Modification of the chemical make-up of PMs can lead to alteration of the release kinetics of encapsulated compounds. There are numerous examples of PMs that release their payload in response to stimuli including alterations in pH^5^,^6^, temperature^7^, oxidation^8^ and light^9^,^10^. This responsiveness can be extended by using variable ratios of blends of different copolymers to tune PM release kinetics^11^. The exquisite control that such technologies can provide has resulted in significant interest in the potential of PMs to probe cell behaviour and enable drug delivery. There are numerous clinical trials being undertaken, predominantly in oncology, which are aiming to achieve better patient outcomes by exploiting the benefits that PM based delivery systems offer.

Polymeric nanoparticles are recognised to have considerable translational potential when considered holistically as drug delivery systems. This approach to disease targeting considers every step of the drug delivery pathway, including reliable scalable PM synthesis, route of administration, biodistribution and pharmacokinetics, through to clearance and excretion^12^. Despite this there remains a need for the quantification of PM uptake by cells and consequent intracellular payload release, which are both essential in determining the pharmacokinetics of drug-delivery and consequent cellular effects. Such information is also important for understanding the concentration of molecules delivered via native carriers, for example, exocytosed vesicles^13^. There is an extensive literature on methods for qualitatively and quantitatively determining the cellular internalisation of nanoparticles. The majority of these methods, however, have relied upon nanoparticles that exhibit intrinsic fluorescence, such as fluorescently labelled PMs^14^ or quantum dots^15^. These methods have been very successful in answering important questions about the kinetics of nanoparticle uptake, but have a number of drawbacks, including an inability to distinguish nanoparticles that are internalised from those that are associated with the external leaflet of cell membranes. Experimental approaches to confirm whether particles are intra-, or peri-cellular have been achieved through the combination of post-hoc correction of measurements collected from flow cytometry and confocal fluorescent analyses^14^,^16^ or by using imaging cytometry^17^. While these approaches are useful for determining uptake of nanoparticles, they still do not provide a method of quantifying intracellular drug release from nanoparticles.

Real-time drug delivery has been investigated previously through the use of fluorescent compounds. Latent fluorophores are molecules that become fluorescent after the release of a quencher^18^,^19^,^20^, or in which the emission and/or excitation wavelength is shifted depending upon chemical dissociation^21^, a cleavage event^22^ or a change in pH (SNARFs)^23^. These fluorescent techniques often require complex coupling chemistries, and they are rarely quantitative. In other work, Battaglia and colleagues have demonstrated that PMs can be used to deliver encapsulated or conjugated dyes to a variety of different cell types with minimal effects on cell viability^24^,^25^,^26^. Further development of these methods would be highly advantageous for quantification of nanoparticle delivery and payload release, particularly when combined with methods that determine whether a delivery molecule is entrapped in a PM or released into the cytosol.

In order to address these challenges, we have exploited some key characteristics of the disodium salt of fluorescein, a water soluble weakly membrane-permeable molecule. Fluorescein has a high quantum yield and an excitation of λ_ex_ 496 nm and emission at λ_em_ 521 nm. However, when fluorescein is highly concentrated in aqueous solution it self-quenches. Self-quenching fluorophores have previously been utilised in the liposome drug delivery field^27^,^28^. These molecules were instrumental in the discovery that some liposome preparations deliver their cargo to cells by endocytosis followed by drug escape from endosomes and early lysosomes, rather than by direct plasma membrane fusion^29^. We hypothesised that encapsulation of fluorescein in the aqueous core of PMs above the quenching concentration would create non-fluorescent nanoparticles carrying a cargo which would fluoresce once released. As PM disruption needs to occur for the fluorophore to be released, we reasoned that this could be used as a tool to quantify the intracellular release of this hydrophilic payload. Fluorescence is observed only when the payload is released and dispersed at a lower concentration within the cell (Fig. 1). We utilize these properties to provide a new method for the quantification of intracellular release of a hydrophilic payload in mammalian cells. L929 fibroblasts were co-incubated with quenched fluorescein-loaded PMs, the time-course and quantity of loaded PMs releasing the payload intracellularly was measured at single cell resolution using flow cytometry and fluorescence spectroscopy.

## Results and Discussion

### Polymersomes stably encapsulate and retain fluorescein at high concentrations

We first tested the hypothesis that PMs are able to carry and stably retain the hydrophilic fluorophore, fluorescein, and that its fluorescence emission would be quenched on high concentration encapsulation in the PM core. PMs were produced by nanoprecipitation^30^ in an aqueous solution containing a high concentration (0.1 M) of fluorescein. In this situation block copolymers self-assemble into vesicular polymersomes, with the aqueous solution retained in their cores.

**Figure 2:**
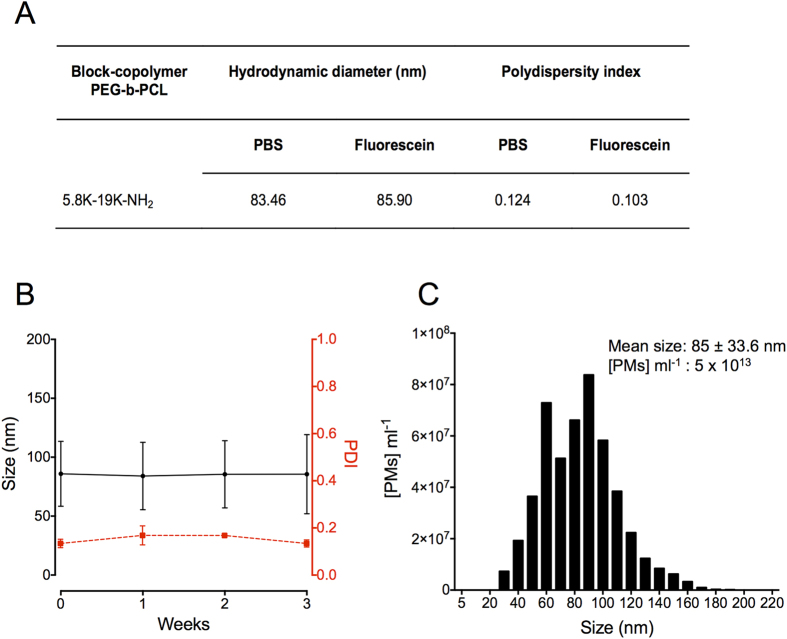
Characterization of PEG-PCL polymersomes (PMs). (**A**) Size and polydispersity index (PDI) of PMs formed from 5.8K-19K-NH2 block-copolymer, measured by dynamic light scattering (DLS). (**B**) Size (black) and PDI (red) stability of 5.8K-19K-NH2 (PEG-b-PCL) fluorescein-loaded PMs over the course of 3 weeks. Data presented as mean ± S.D. (**C**) Histogram depicting size and concentration per ml of fluorescein-loaded PMs measured by nanoparticle tracking analysis (NTA).

PMs were formed from 5.8K-19K-NH_2_ block copolymers. In previous work, we have produced PEG-PCL PM with this class of block copolymer, and we demonstrated the functionality of these PMs as a payload carrier *in vivo*^31^ but as yet we have not examined its cellular uptake by cells, *in vitro* or *in vivo*. After fluorescein encapsulation, no significant changes were found in hydrodynamic radius or polydispersity, which is a measure of the width of the particles size distribution and which is considered to be less than 0.2 for homogeneous preparations^32^ (Fig. 2A), and 5.8K-19K-NH_2_ PMs were stable over a period of 3 weeks, with no measurable change in either of these variables (Fig. 2B).

Nanoparticle tracking analysis (NTA), a technique that enables the visualisation, sizing and quantification of nanoparticles in suspension by the use of a highly sensitive video camera^33^, revealed a hydrodynamic diameter value of 85 ± 33.6 nm for 5.8K-19K-NH_2_ PMs, in close agreement with the value obtained by dynamic light scattering (DLS) (hydrodynamic diameter of 86 nm and PdI of 0.103). NTA analysis determined the PM concentration to be 5.0 × 10^13^ nanoparticles ml^−1^ (Fig. 2C). This type of measurement allows for a precise quantification of the final PM concentration, overcoming calculations purely based on assumptions of PM physical characteristics (i.e. membrane thickness, area of the molecules and copolymer weight)^14^,^24^.

**Figure 3:**
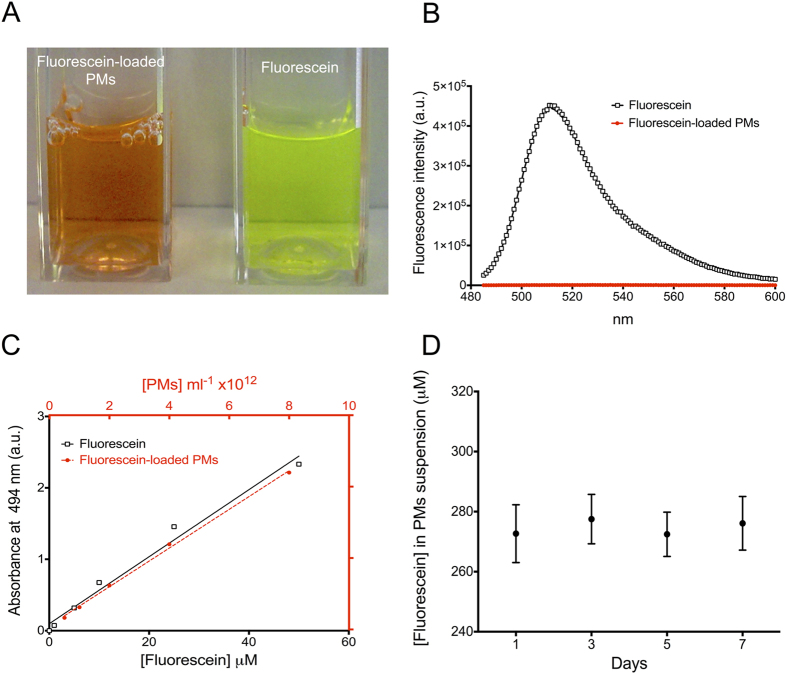
High concentration packaging of fluorescein in PMs quenches fluorescence. (**A**) Picture showing the difference in color of fluorescein-loaded PMs and fluorescein at equal concentrations. (**B**) Absence of fluorescence emission from fluorescein-loaded PMs (red) compared to equivalent concentration of free fluorescein (274 μM) (black) measured by spectrophotometry. (**C**) Absorbance measurements (at 494 nm) of increasing concentrations of fluorescein-loaded PMs and free fluorescein. (**D**) Retention of fluorescein in loaded-PMs over the course of one week measured by absorbance (at 494 nm). Data presented as mean ± S.D. of three independent measurements.

Encapsulation of fluorescein (0.1 M), followed by dialysis of the PMs and size exclusion chromatography, resulted macroscopically in a PM suspension that was dark orange in colour, with none of the characteristic yellow appearance of fluorescein in solution (Fig. 3A). Confirming this, no detectable fluorescence was found in the PM suspension by fluorescence spectroscopy when compared to equivalent concentration of fluorescein, indicating quenching of the PM-encapsulated fluorescein and absence of free fluorescein (Fig. 3B). The concentration of fluorescein in PM preparations was measured using absorbance spectroscopy. A linear relationship was found between PM concentration and absorbance at concentrations <50 μM (Fig. 3C). Fitting of absorbance measurements of PMs to an absorbance standard curve for fluorescein, allowed us to determine a concentration of 273 ± 7.4 μM fluorescein in PMs at a concentration of 5 × 10^13^ PMs ml^−1^. From this, we calculate a mass of 5.48 × 10^−21^ moles of fluorescein per PM. Note that there is no absorbance interference from the block copolymer at this wavelength (no significant difference between absorbance of PM suspension compared to PBS background reading at 494 nm). Furthermore, note that the absorbance curve for fluorescein is linear between 0 and 50 μM, but becomes non-linear due to complete absorbance at high concentrations. All measurements were made in the linear range of absorbance for fluorescein^34^.

The concentration of fluorescein inside the PM aqueous core was stable over the course of a week (Fig. 3D), indicating that there was no significant loss of internalised fluorescein from the PMs, despite the high concentration gradient across the PM shell membrane following dialysis and storage in aqueous solvent. Confirming this, only very low concentrations of free fluorescein (1 μM ± 0.1 μM) were found in the supernatant of ultracentrifuged polymersome preparations (See Supplementary Fig. S1). Together these experiments demonstrate that PMs trap fluorescein and that its fluorescence is quenched at the concentrations present inside the PMs^27^,^35^. The physical characteristics of the PMs are unaffected by the inclusion of fluorescein, and the PMs retain fluorescein stably without significant leakage over prolonged time periods at room temperature.

In previous studies, a similar method was used to incorporate fluorescein and its derivative carboxyfluorescein in liposome carriers. In these studies, liposomes were found to be permeable to fluorescein, but relatively impermeable to carboxyfluorescein (which differs from fluorescein by an extra carboxyl group at the 5- and 6-position, making it more hydrophilic)^27^,^36^. PMs are known to be less permeable for a variety of molecules compared to liposomes (for example they are an order of magnitude less permeable to water than liposomes)^37^, and the current data suggests that this is true for fluorescein, with insignificant leakage even after a period of several days.

### Fluorescein-loaded PMs are actively taken up by mammalian cells and subsequently release their payload intracellularly

**Figure 4:**
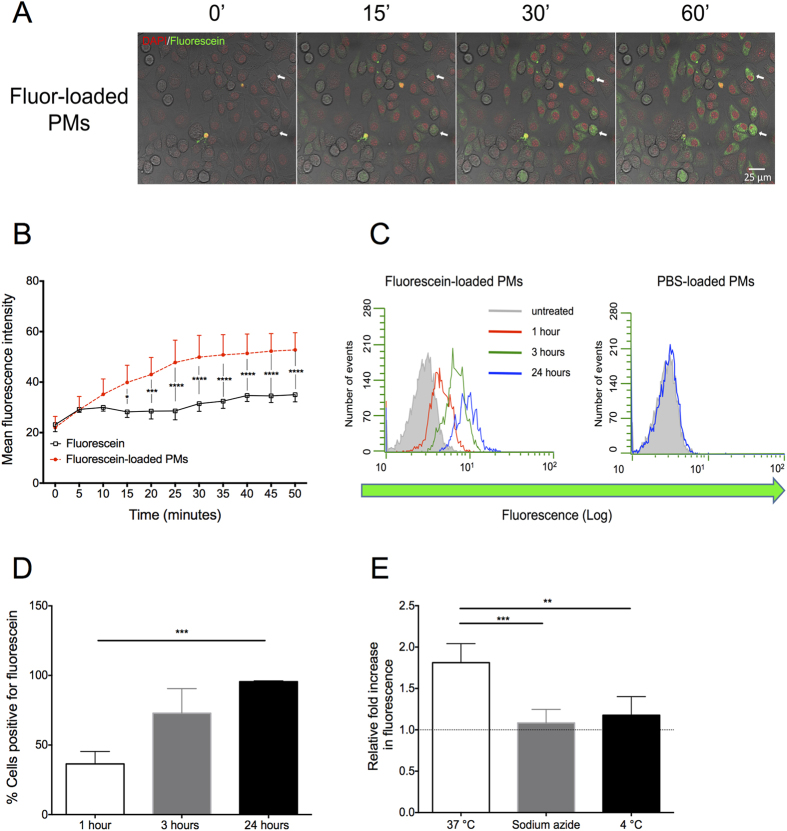
Fluorescein-loaded PMs are actively taken up by mammalian cells and subsequent release their payload intracellularly. (**A**) Time-lapse imaging of L929 cells incubated with fluorescein-loaded PMs shows time-dependent intracellular release of fluorescein. Images were taken up to 1 hour after the addition of the fluorescein-loaded PMs and are a projection of a single z-stack. Green: cells positive for fluorescein. Red: nuclear staining. White arrows indicate two cells showing a change in fluorescence across the experimental time-course. (**B**) Image analysis of the mean fluorescence intensity of L929 cells incubated with fluorescein-loaded PMs (dot) or fluorescein (square) for one hour. The results show a steady and significant increase in cells exposed to PMs as opposed to un-encapsulated fluorescein. Data presented as mean ± S.D. Statistical analysis is two-way ANOVA. (**C**) Representative flow cytometry histogram plot of L929 cells incubated for 1 (red), 3 (green) or 24 hours (blue) with either fluorescein-loaded PMs or PBS-loaded PMs. (**D**) Percentage of cells positive for fluorescein after 1, 3 or 24 hours incubation with fluorescein-loaded PMs. Data presented as mean ± S.D. Statistical analysis is Kruskal-Wallis one way ANOVA on ranks followed by Dunn’s post-hoc test. (**E**) Relative levels of fluorescence of L929 cells incubated with fluorescein-loaded PMs for 5 minutes under 3 conditions: at 37 °C, at 4 °C and at 37 °C with sodium azide. Fluorescence is normalized against autofluorescence from control cells where no fluorescein-loaded PMs were added (dashed line). Data presented as mean ± S.D. Statistical analysis is one way ANOVA followed by Tukey’s post-hoc test. *p < 0.05, **p < 0.01, ***p < 0.001, ****p < 0.0001.

To determine whether PMs are taken up and their contents are released in mammalian cells, fluorescein-loaded PMs were added in solution to cultures of the mammalian fibroblast cell line, L929. Fibroblasts are known to be pivotal in contributing to the progression of several malignancies including endometrial cancer^38^, and therefore represent a possible target for nanoparticle-based therapeutic approaches. Following co-incubation of cell-cycle synchronised (serum-starved) L929 cells with fluorescein-loaded PMs, a time-dependent increase in the fluorescence of cells from approximately 15 minutes after the start of the incubation was observed, as seen in confocal images (Fig. 4A). This indicates uptake of PMs and intracellular release of fluorescein. Quantification of cellular fluorescence revealed a gradual increase over a period of one hour, with evidence of significantly higher intracellular payload release than that measured in controls, incubated with free (un-encapsulated) fluorescein, within 15 minutes (p < 0.05) (Fig. 4B) of the start of the experiment. These results indicate that PMs loaded with a high concentration of fluorescein release their cargo intracellularly, and that fluorescence of the dye molecules is no longer quenched on release. Encapsulated dyes such as rhodamine B and propidium iodide, were shown to be released intracellularly from polymersomes following uptake^24^. The polymersomes studied were fabricated to include a pH-sensitive poly (2-(di-isopropylamino) ethylmethacrylate) (PDPA) block. On exposure to a pH ~6.5, equivalent to lysosomal pH of, the PDPA underwent dissociation of the di-block polymers, leading to release of encapsulated agents in short (ms) timeframes. PCL-PEG co-block polymer is less pH sensitive, and undergoes hydrolysis and degradation over relatively longer time frames^11^,^39^. It is therefore somewhat surprising that we measured dye release under such short time frames (minutes). We suggest that, due to the presence of PCL which is a highly hydrolase-sensitive polyester^40^, the release in our experiments may be facilitated by lysosomal acid hydrolases after initial partitioning of PM in early endosomes and lysosomes. Hydrolysis of polyesters, which induces the destabilisation of the vesicle structure is likely leading to the accelerated release of fluorescein at physiological temperature (~37 °C) compared to the storage temperature of the PM preparation (room temperature)^41^. Further high-resolution imaging studies would enable better characterisation of this process. Additionally, it is likely that the observed fluorescence release originates from only a proportion of internalised PMs (as the fluorescence of fluorescein in intact PMs will remain quenched). Future studies may address this by comparing fluorescence release of quenched dyes in, for example, pH sensitive PMs compared to more environmentally robust PMs.

To quantify the rate and magnitude of PM-mediated fluorescein release in cultured cells on a cell-by-cell basis, we used flow cytometry. As in the confocal microscopy experiments, time-dependent increases in fluorescence were detected in populations of cells exposed to PMs. After 1h, 36 ± 9% of cells showed increases in fluorescence above that of any cell co-incubated with a PBS-loaded control, and 95.4 ± 0.6% of cells after 24 hours. This suggests that nearly all cells in the treated populations had taken up PMs and undergone intracellular release of PM-encapsulated fluorescein over this time period (Fig. 4C,D; note 4D is data obtained from 3 separate experiments; data from one experiment is shown in the cytometry plot in 4C).Time periods of exposure for longer than 24 hours did not result in any further uptake (Supplementary Fig. S2). Uptake of PMs had no significant effect on cell viability (See Supplementary Fig. S3), in agreement with other published studies on similar PMs^25^. Confirming our earlier confocal observations, there was also significant heterogeneity in fluorescein release within the cell populations, despite the cell cycle being synchronised (note the variability in fluorescence in the cells shown in the images captured by confocal microscopy and in the flow cytometry histograms Fig. 4A,C respectively).

Next we tested the hypothesis that PM uptake is an active, energy-dependent process. To achieve this, we assayed uptake at low temperature and after treatment with the ATP synthase inhibitor sodium azide. The relative increase in intracellular fluorescence of L929 cells incubated for 5 minutes with fluorescein-loaded PMs was significantly higher at 37 °C compared to cells incubated at 4 °C (p < 0.01), or those treated with the mitochondrial energy inhibitor sodium azide (p < 0.001) (Fig. 4E). Negligible cellular fluorescence was found after incubation of L929 cells with fluorescein at a concentration equivalent to that measured as a residual, un-encapsulated, concentration found in all PM preparations (~1 μM, See Supplementary Fig. S4), excluding a potential passive uptake of fluorescein from the culture medium. Together, these results illustrate that PEG-PCL PMs are quickly internalised by mammalian cells, in a time-dependent manner, do not induce cytotoxicity, and that this process is an active and energy-dependent process, in agreement with previous reported findings^14^,^31^,^42^,^43^,^44^,^45^.

Our experimental setup did not allow for the distinction between the different energy-dependent mechanisms of endocytosis (clathrin-mediated endocytosis, macropinocytosis, caveolae-mediated endocytosis and clathrin and caveolae-independent endocytosis). Nevertheless, our results corroborate findings from Xin *et al*. who demonstrated that PEG-PCL nanoparticles, which display a specific receptor-binding motif (Angiopep-2) on their surface are internalised by cells *via* energy dependent mechanisms, in particular caveolae-mediated endocytosis^44^. While the use of inhibitors of specific pathways of endocytosis (e.g. sodium azide, filipin and cytochalasin D) allow for a more detailed characterisation of the internalisation process, they may also impair the physiological cellular response inducing preferential cellular uptake *via* specific pathways. Chernenko *et al*. demonstrated the compartmentalisation of PEG-PCL nanoparticles in secondary endosomes of Hela cells by following the vibrational signature of PCL molecules and their released cargo using label-free Raman imaging^46^,^47^. The ability to distinguish specific routes of cellular uptake would be beneficial in defining the sub-cellular partitioning of either PMs and payload and could be investigated in future experiments, but the quantification of payload released intracellularly is equally important.

### Intracellular payload release can be quantified at single-cell resolution, revealing heterogeneity in cellular delivery

**Figure 5:**
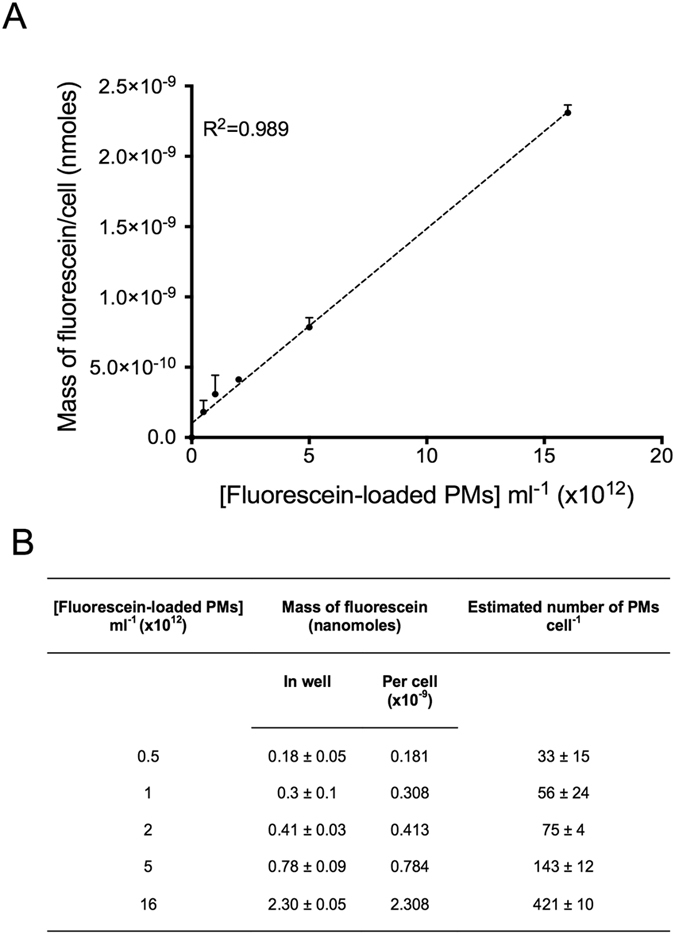
The intracellular mass of fluorescein is proportional to extracellular PM concentration. (A) Graph depicting the linear relationship between the mass of fluorescein per cell and the concentration of fluorescein-loaded PMs incubated with the cells. R^2^ = 0.989. Data presented as mean ± S.D. (**B**) Mass of fluorescein per well and per cell, as well as estimated number of PMs per cell following 24 hour incubation with increasing concentrations of fluorescein-loaded PMs. Data presented as mean ± S.D.

In order to determine the amount of fluorescein delivered intracellularly, a defined number of cells were pre-incubated with PMs for 24 hours. Cells were washed thoroughly with PBS in order to remove excess PMs present in solution but not internalised by cells, and were then lysed and the concentration of intracellular fluorescein was measured using a standard curve for fluorescence. The quantum yield of fluorescein is known to be affected by its molecular nanoenvironment (eg pH, charge, protein binding)^34^. In order to control for these factors, the standard curve was determined by measurement in a solution equivalent to that produced by lysed cells (See Supplementary Fig. S5). A linear relationship was found between the initial concentrations of PMs incubated with cells and the amount of fluorescein recovered from within the cells after 24 hours (Fig. 5A). In addition, we found no difference in the total intensity of aliquots of cells before and after cell lysis (See Supplementary Fig. S6), indicating that the quantum yield of fluorescein is relatively unaffected by its molecular microenvironment following intracellular release. This is somewhat surprising as it is known that decreasing the pH leads to a decrease in the quantum yield of the fluorophore^48^. Since some data suggest the partitioning of PMs into endosomes and lysosomes^46^, where pH can range from 6.5 for endosomes to 5 for lysosome, it may be the case that in this study, fluorescein is largely partitioned in early endosomes or in the cytoplasmic space, where the pH is higher. Longer periods of incubation (>24 hours), with an associated increase in the lysosomal partitioned cargo would be predicted to cause reductions in the measured intracellular quantum yield of fluorescein in comparison to post-lysis. Despite this, we cannot rule out the possibility that our estimations of intracellular fluorescein may be subject to error. As fluorescein may be in various stages of release and therefore at heterogeneous concentrations within the cells, and as our methods of cell disruption may not completely disrupt intracellular organelles including endosomes and lysosomes, the recovered fluorescence yield may be an underestimation of true intracellular fluorescein concentration. Other methods, such as high performance liquid chromatography, may be used in the future to provide an additional layer of certainty of the absolute intracellular concentration of the dye. Notably, Gottstein *et al*. used a correction factor to normalise the intracellular solid fluorescence of polystyrene nanoparticles to their external fluorescence^16^. While this is an elegant method for determining the intracellular uptake of cohesive, solid fluorescent capsules, it would be more difficult to employ this technique in the current study. In Gottstein *et al.’ s* report, the nanoparticles are not subject to changes in the local molecular concentration through diffusion and are less subject to intermolecular interactions since most of the nanoparticles are labelled with the fluorophores and this is not contained within the particles. Future studies may address local changes in the microenvironment in combination with measurements of concentration by fluorescence. This may be achieved by, for example, pH sensitive dyes, such as SNARFs employed by Semmling *et al*.^23^. Or alternatively, time resolved fluorescence could be used to determine the relative concentrations of a fluorophore in one molecular microenvironment compared to another. In previous studies, this technique has been used to measure the relative amount of fluorophores in mitochondria compared to cytosol^49^,^50^.

At 24 hours, cells incubated with the concentration of PMs used in Fig. 4 (5 × 10^12^ PMs ml^−1^) contained 7.85 × 10^−10^ nanomoles of fluorescein *per* cell (Fig. 5B). Based on our earlier calculations, the mass of fluorescein in a PM is 5.48 × 10^−21^ moles, enabling the number of nanoparticles releasing their cargo in cells to be calculated using the following formula:


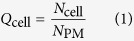


where Q_cell_ is the average number of PMs releasing their payload *per* cell, N_cell_ is the number of moles of fluorescein *per* cell and N_PM_ is the number of moles of fluorescein *per* nanoparticle. Calculations are presented in Fig. 5B. At a PM concentration of 5 × 10^12^ ml^−1^, this indicates that an average number of ~143 ± 12 PMs are internalised and release their contents *per* cell over a period of 24 hours (Fig. 5B).

**Figure 6:**
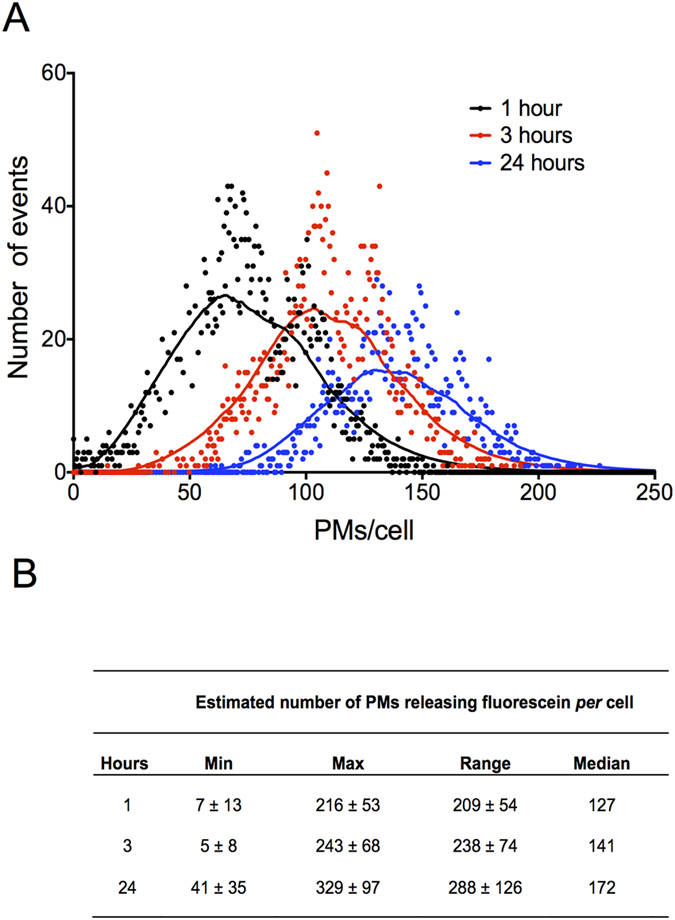
Intracellular payload release can be quantified at single-cell resolution and reveals heterogeneity in cellular delivery (**A**) Estimated number of PMs internalized per cell after 1 hour (black), 3 hours (red) or 24 hour (blue) incubation with fluorescein-loaded PMs. Data is representative of a single experiment. (**B**) Estimated minimum (Min), maximum (Max), range (Max-Min), median and mean number of PMs per cell after 1, 3 or 24 hours incubation with fluorescein-loaded PMs.

Fluorescence spectroscopy only provides an average quantification of fluorescein release from PMs, therefore flow cytometry was used to determine cell-specific nanoparticle payload release, and the inherent degree of variability in the cellular population. By normalising fluorescence intensity data from flow cytometric analysis to measurements of intracellular PM release obtained by fluorescence spectroscopy (above), the PM payload release was calculated for every cell in a flow cytometric analysis at 1, 3 and 24 hours post addition of PMs (Fig. 6A). As expected, we measured an increase in the mean intracellular release with respect to time^14^,^24^, but also an increase in the variability of fluorescein load in the cellular population. This was despite serum starvation, which synchronises the cell cycle, prevents cell division and reduces intra-population uptake variability^51^. The observed cell-to-cell variability in PM release of fluorescein may be attributed to the stochastic nature of cell-PM interactions during the internalisation process, resulting from a combination of factors including PM agglomeration and clustering and variable cellular surface receptor dynamics^52^,^53^. In addition, our analysis assumes that PMs of all sizes have an equal chance of being taken up and releasing their contents per cell, regardless of size. This may be an over simplification, as size is known to affect the rate and efficiency of uptake^24^,^54^. Since the mass of fluorescein that each PM contains is proportional to volume rather than diameter, measurements of uptake may be particularly sensitive to variations in the uptake of PMs at the large end of the dispersion profile, This observation underlines the need for further quantitative analysis of putative drug release at a single-cell resolution.

Single cell analyses demonstrated a time-dependent increase in the maximum number of PMs releasing their contents intracellularly *per* cell (217 ± 53, 243 ± 68 and 329 ± 35 PMs *per* cell after 1, 3 and 24 hour incubations respectively). This reflects a wider range (Max PMs *per* cell – Min PMs *per* cell) of PMs internalised after different incubation periods and describes an incremental heterogeneity in PM dye release within the cellular population (Fig. 6B). Furthermore, in the single cell analysis, the estimated median value of PMs *per* cell after 24 hour incubation (~173 ± 38) is very similar to the values calculated from the fluorescence spectroscopy quantification (~143 ± 12) (Fig. 5B). Comparing the number of nanoparticles internalised *per* cell quantified in different studies is fraught with difficulty, considering the diversity of cells, nanoparticles and cell culture conditions used. Nevertheless, very recently Unciti-Broceta *et al*. estimated the number of nanoparticles needed *per* cell in order to achieve nanoparticle uptake in 50% of a cell population^55^. The figure estimated by the authors for 3 different cell lines are in the same order of magnitude to the number of nanoparticles *per* cell calculated here and similar to previous reports^16^,^56^. In another study, Massignani *et al*. recently used either the encapsulation, or conjugation, of rhodamine B to diblock polymers composed of PMPC25–PDPA70 and PEG23–PDPA15 diblock copolymers^24^. The authors reported values of 10^6^–10^7^ PMs internalised/cell, several orders of magnitude higher than our current study. This difference is striking, but can be partially explained by the higher concentration of PMs used in this study (10^16^/mL), differences in the PM chemistry and encapsulation efficiency of the dye molecules used, and finally differences in the degradability of the PMs used – in our study, we quantified only the amount of fluorescein that was released from PMs, and we did not measure dye which remained quenched within intact PMs.

The quantification method described here could be extended to different PM preparations and importantly enables single cell analysis to be performed. Another factor that influences the kinetics of PM uptake and subsequent intracellular release is potential PM depletion from the culture medium, particularly when nanoparticle uptake in a given time period is comparable with the number of nanoparticles available *per* cell^17^,^57^. In a recent study using solid PEG-conjugated polystyrene nanoparticles however, Unciti-Broceta *et al*. found that a ratio of 1000–4000 nanoparticles per cell was saturating, with nanoparticle concentration becoming rate limiting only below 2000 nanoparticles per cell^55^. In our experiments we calculate a concentration of >1 × 10^6^ PMs*/*cell, and so it is unlikely that the depletion of nanoparticles from the medium has any significant effect on the rate of uptake.

## Conclusion

All drug-delivery systems must undergo extensive characterisation prior to evaluation in clinical trials^58^. There is evidence from *in-vivo* studies in animals that delivery using polymeric nanoparticle preparations has clear advantages over administration of free drug, with fewer off-target and toxic effects^59^. Studies correlating preclinical findings and data from human trials have found that there is good correlation between the biodistribution and clearance of the particles between these two study types^60^ and several polymeric nanoparticle delivery systems, e.g. Genexol-PM, are now in phase 2 clinical trials^61^. However a current limitation with the use of polymersomes (PMs) for controlled drug delivery is the paucity of information regarding the number of nanoparticles internalised at a single cell level and therefore the concentration of payload released. We exploited the characteristics of sodium fluorescein to demonstrate real-time delivery of a hydrophilic compound in a mammalian fibroblast cell line using PMs. To our knowledge, this is the first attempt to quantify the intracellular release of a fluorophore from a hollow polymeric nanocarrier at a single cell level. When nanoparticles are considered as possible delivery vehicles for therapeutic molecules at a cellular and sub-cellular level, the heterogeneity of distribution within a population is a critical factor that should be considered. The simple methodology proposed here could be extended to quantify the cellular internalisation of PMs formed from different materials, including stimuli responsive PMs. Additionally, this method could be used to determine the relative partitioning of a putative drug in populations of mixed cell types, as will invariably be the case in *in vivo* studies, and how drugs partition during cell division^17^. It provides a methodology with which to better understand how the kinetics of cellular uptake might be affected by cell type, nanoparticle polymer chemistry, and the addition of nanoparticle surface moieties. We believe that these types of investigations are much needed in order to fully exploit the potential of nanoparticles, particularly PMs for clinical applications.

## Material and Methods

### PM preparation

The following methodology was adapted from previously reported work^30^. PMs were prepared by dissolving α-amino-ω-hydroxy terminated (NH_2_)-PEG-b-PCL (6.0 mg) (Polymer Source, Dorval, Canada) in dimethylformamide (DMF) (0.4 ml). The amphiphilic copolymer used had an average molar mass (M_n_) of 5.8 × 10^3^ g/mol for the PEG block and 19 × 10^3^ g/mol for the PCL block. The solution was then placed in an ultrasonic bath to aid dissolution. A 0.1 M sodium fluorescein (Sigma-Aldrich, Poole, UK) solution (10% DMF in 0.1 M phosphate buffer (PBS)) in PBS was prepared. The polymer solution was then added drop-wise (~1 drop every 8 seconds) to the sodium fluorescein (1.60 mL) with rapid stirring. The sample was then dialyzed (regenerated cellulose, 10,000 MWCO, Sigma-Aldrich, Poole, UK) against PBS (400 mL), replacing the buffer solution 4 times over the course of 48 hours. Fluorescein-loaded PMs were then removed from the dialysis tubing and filtered through a size separation column (Sephadex G-25, Sigma-Aldrich, Poole, UK) to remove any remaining un-encapsulated fluorescein.

### Dynamic light scattering (DLS)

DLS analysis (Zetasizer Nano ZS ZEN3600, Malvern Corp, Worcestershire, UK) was performed by diluting PMs (100 μl) in MilliQ PBS (1900 μl). Prior to the analysis, samples were filtered into a plastic cuvette through a cellulose acetate syringe filter (0.2 μm) (Minisart, Sigma-Aldrich, Poole, UK). Measurements were completed at 20 °C with the light detected at a scattering angle of 173°. Data was collected 13 times/sample. Acquisition was for 5 seconds and each measurement was carried out in triplicate.

### Nanoparticle tracking analysis (NTA)

A NanoSight device (LM10, Malvern Corp, Worcestershire, UK) was used to measure both PMs hydrodynamic size and concentration. PMs were diluted (1 μl) in MilliQ PBS (100 ml), filtered and loaded in the detection cell prior to measurement.

### Fluorescence intensity

Fluorescein-loaded PMs and solutions of fluorescein of equivalent concentration (274 μM) were analysed for fluorescence intensity over a range of wavelengths (increment of 1 nm from 485 nm to 600 nm) using a FluoroMax-4 (Horiba Scientific, Stanmore, UK). Release of fluorescein into supernatant after storage was also measured using a FluoroMax-4 (Horiba Scientific, Stanmore, UK).

### Fluorescein encapsulation and release *in vitro*

The concentration of sodium fluorescein was calculated by measuring the UV absorbance (Nanodrop 2000c, Thermo Fisher Scientific, Basingstoke, UK) compared to stock solutions. To determine the kinetics of sodium fluorescein release, PM samples were kept at room temperature (20 °C) under constant dialysis (regenerated cellulose, 10,000 MWCO, Sigma-Aldrich, Poole, UK) against PBS. At various intervals over a total of 7 days PM samples were collected. 1 mL aliquots of each sample was then passed through a size separation column, before measurement of UV absorbance (Nanodrop 2000c, Thermo Fisher Scientific, Basingstoke, UK). Each experiment was performed in triplicate. All of the preparations give a 3 mg/mL solution of PMs. For all *in vitro* experiments PM samples were used immediately and were sterile filtered through a 0.2 μm cellulose acetate syringe filter before use.

### Cell culture

Mouse L929 Fibroblasts (ATCC) were maintained in Dulbecco’s Modified Eagle medium (DMEM) (Thermo Fisher Scientific, Basingstoke, UK) supplemented with 10% (V/V) fetal bovine serum (FBS) (Thermo Fisher Scientific, Basingstoke, UK), 100 U/ml of penicillin (Thermo Fisher Scientific, Basingstoke, UK) and 100 μg/ml of streptomycin (Thermo Fisher Scientific, Basingstoke, UK). The cells were incubated at 37 °C and 5% CO_2_. For fluorescence microscopy and cytofluorimetric analysis, 24 hours before an experiment the medium was replaced with DMEM supplemented with low serum (0.5% V/V), 100 U/ml of penicillin (Thermo Fisher Scientific, Basingstoke, UK) and 100 μg/ml of streptomycin (Thermo Fisher Scientific, Basingstoke, UK).

### Confocal microscopy

L929 cells were seeded at a density of 50,000 cells/cm^2^. The medium was replaced with DMEM supplemented with low serum 24 hours before the experimental assay. 2 hours before PM addition, the medium was replaced with piperazine-N,N′-bis (2-ethanesulfonic acid) (PIPES) buffer supplemented with 0.5% serum. Fluorescein-loaded PMs were added (5 × 10^12^ PMs ml^−1^) to the cells and images were taken every 60 seconds over 60 minutes using a TCS SP5 laser scanning confocal microscope (Leica, Milton Keynes, UK). Cell nuclei were stained with Hoechest 33342 (Thermo Fisher Scientific, Basingstoke, UK). A 63 × 1.30 glycerol immersion objective was used. Negative controls were, PBS-loaded PMs (5 × 10^12^ PMs) and a 10 mM solution of fluorescein. Image analysis was performed by randomly selecting 20 cells of interest and measuring the mean fluorescence intensity at each time point.

### Energy-dependent internalization

L929 cells were seeded at a density of 50,000 cells/cm^2^. The medium was replaced with DMEM supplemented with low serum 24 hours before the experimental assay. PMs were added to the cells (5 × 10^12^ PMs ml^−1^) and incubated for 5 mins, after which the medium was removed and replaced with fresh DMEM supplemented with low serum. After 1 hour the cells were washed x3 with ice-cold PBS, fixed in 4% paraformaldehyde for 15 minutes and imaged using an Axiovert200 inverted microscope (Zeiss, Birmingham, UK). To inhibit energy dependent processes, sodium azide (1 mg/ml) was added for 20 minutes prior to adding fluorescein-loaded PMs. For experiments at 4 °C, cells were equilibrated by keeping them on ice for 30 minutes prior to adding fluorescein-loaded PMs. The medium, PBS and 4% paraformaldehyde were kept on ice. Cells were fixed for 20 minutes. Using Image J software line scans of the width of the cell were taken from the images to give the mean fluorescence *per* cell. Relative fluorescence was calculated normalizing against the auto-fluorescence of the control.

### Cytofluorimetry

L929 cells were seeded in 6 well plates at a density of 1 × 10^4^ cells/well. The medium was replaced with DMEM supplemented with low serum 24 hours before the experimental assay. PMs were added to the cells (5 × 10^12^ PMs ml^−1^) and incubated for 1, 3 or 24 hours. Medium was removed at the designated time points, cells were washed using PBS, detached using trypsin and re-suspended in 500 μl of PBS. For propidium iodide (PI) (Sigma-Aldrich, Poole, UK) staining, a solution of 2 mg/ml (1 μl) was added to each sample just before cytofluorimetric analysis (Guava easyCyte, Millipore, Milton, UK).

### Quantification of the number of PMs per cell

L929 cells were seeded in a 6 well plate at a density of 100,000 cells/well and incubated for 24 hours with increasing concentrations of fluorescein-loaded PMs (from 5 × 10^10^ to 2 × 10^12^ PMs ml^−1^). Then, cells were washed with PBS twice to remove the excess of PMs that were not internalised and lysed using a solution of HEPES (20 mM), NaCl (125 mM) and sodium dodecyl sulphate (SDS) (2%). The relative optical density (O.D.) was measured at 521 nm using a Varioscan Flash microplate reader (Thermo Fisher Scientific, Basingstoke, UK). A fluorescein calibration curve was produced by dilution of a stock solution of fluorescein (100 mM) (Sigma-Aldrich, Poole, UK) in PBS supplemented with cell lysate. Mass of fluorescein PM^−1^ was calculated as the ratio between the mass of fluorescein present in PMs solution by the concentration of PMs ml^−1^. For quantification at the single cell level, raw data was extracted from FCS files using Matlab (Mathworks, Cambridge, UK) and analysed using Microsoft Excel.

### Statistical analysis

The data was tested for normal distribution using the D’Agostino-Pearson normality test using graph pad prism software (GraphPad Software, La Jolla, USA). Parametric tests were carried out for normally distributed data, whereas non-parametric tests were used for skewed data. Comparisons between different treatments were made using one-way or two-way ANOVA, followed by either Tukey’s or Dunn’s post hoc test. Statistical significance was defined as *p < 0.05, **p < 0.01, ***p < 0.001, ****p < 0.0001 and n.s. p ≥ 0.05.

## Additional Information

**How to cite this article**: Scarpa, E. *et al*. Quantification of intracellular payload release from polymersome nanoparticles. *Sci. Rep.* **6**, 29460; doi: 10.1038/srep29460 (2016).

## Supplementary Material

Supplementary Information

## Figures and Tables

**Figure 1:**
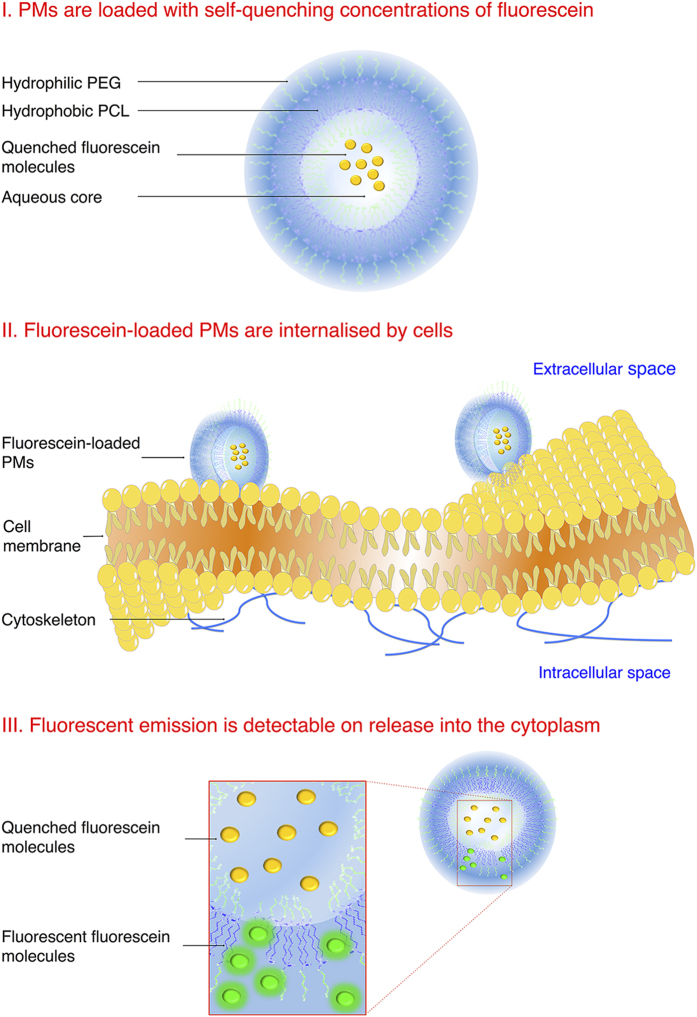
The principle of use of quenched fluorescein to measure intracellular payload delivery (I) PEG-b-PCL block copolymers self-assemble in an aqueous environment to form polymersome structures. The PCL units of the polymer form a hydrophobic membrane, the PEG units form a hydrophilic corona and line the interior cavity of the polymersome. (II) When carrying a payload of sodium fluorescein above the quenching concentration the polymersome is non-fluorescent. Upon cellular internalisation (III) payload release can be visualized as the fluorescein disperses and falls below the quenching concentration.
