# Supplementary material for: Quantification of intracellular payload release from polymersome nanoparticles
Source: Sci Rep. 2016 Jul 11;6:29460. doi: 10.1038/srep29460 (PMC4941396; doi:10.1038/srep29460)
Supplement: Supplementary Information [file srep29460-s1.pdf]

Quantification of intracellular payload release from polymersome nanoparticles

*Edoardo Scarpa[1,2], Joanne L. Bailey[2], Agnieszka A. Janeczek[1,2], Patrick S. Stumpf[1,2], Alexander H. Johnston[2], Richard O. C. Oreffo[1,2], Yin L. Woo[3,4], Ying C. Cheong[1], *Nicholas D. Evans[1,2,5], *Tracey A. Newman[2,6]*

[1]Centre for Human Development, Stem Cells and Regeneration, University of Southampton Faculty of Medicine, Tremona Road, Southampton, SO16 6YD, United Kingdom. [2] Institute for Life Sciences, Centre for Biological Sciences, B85, University Road, University of Southampton, [3] Department of Obstetrics and Gynaecology, Faculty of Medicine, University of Malaya, Kuala Lumpur, 50603, Malaysia.[4]University of Malaya Cancer Research Institute (UMCRI), University of Malaya, Kuala Lumpur, 50603, Malaysia. [5]Bioengineering Sciences Group, Faculty of Engineering and the Environment, University of Southampton, Highfield, Southampton, SO17 1BJ, [6]Clinical and Experimental Sciences, Medicine, University of Southampton, SO17 1BJ, United Kingdom

*Corresponding authors: Tracey Newman tan@soton.ac.uk; Nicholas Evans: n.d.evans@soton.ac.uk

Supplementary Figure 1

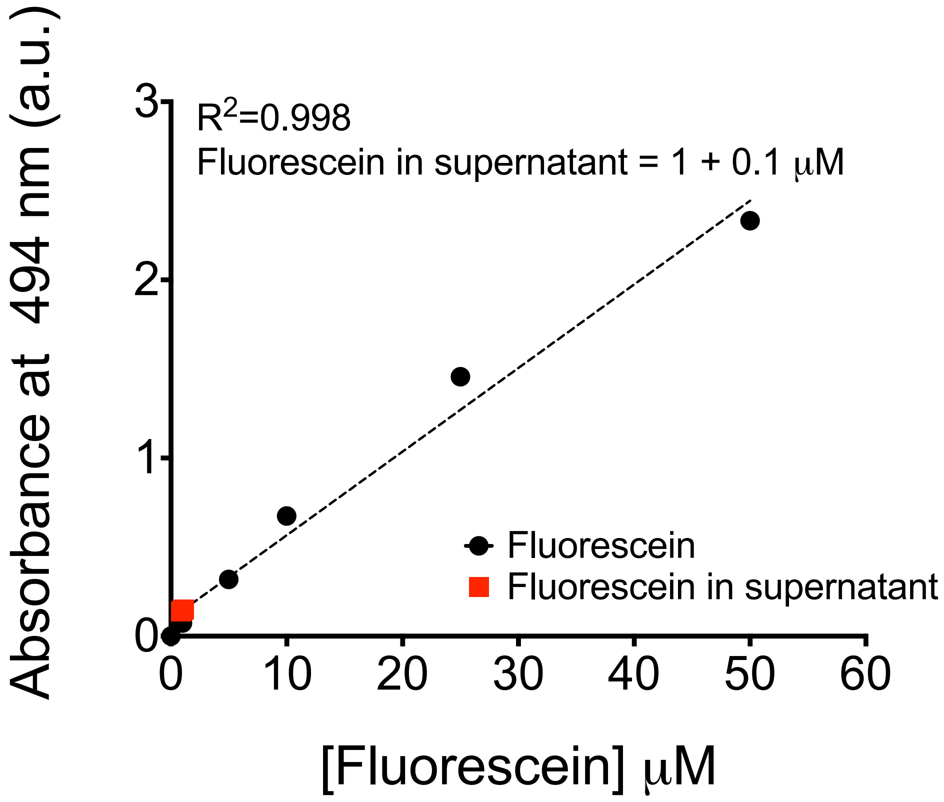

Figure S1. Low concentrations of free fluorescein present in PMs preparation. Following preparation, fluorescein-loaded PMs were ultracentrifuged for 1h at 100,000 g, the supernatant was collected and its absorbance was compared against a standard curve of increasing concentrations of fluorescein. Data presented as mean ± S.D.

Supplementary Figure 2

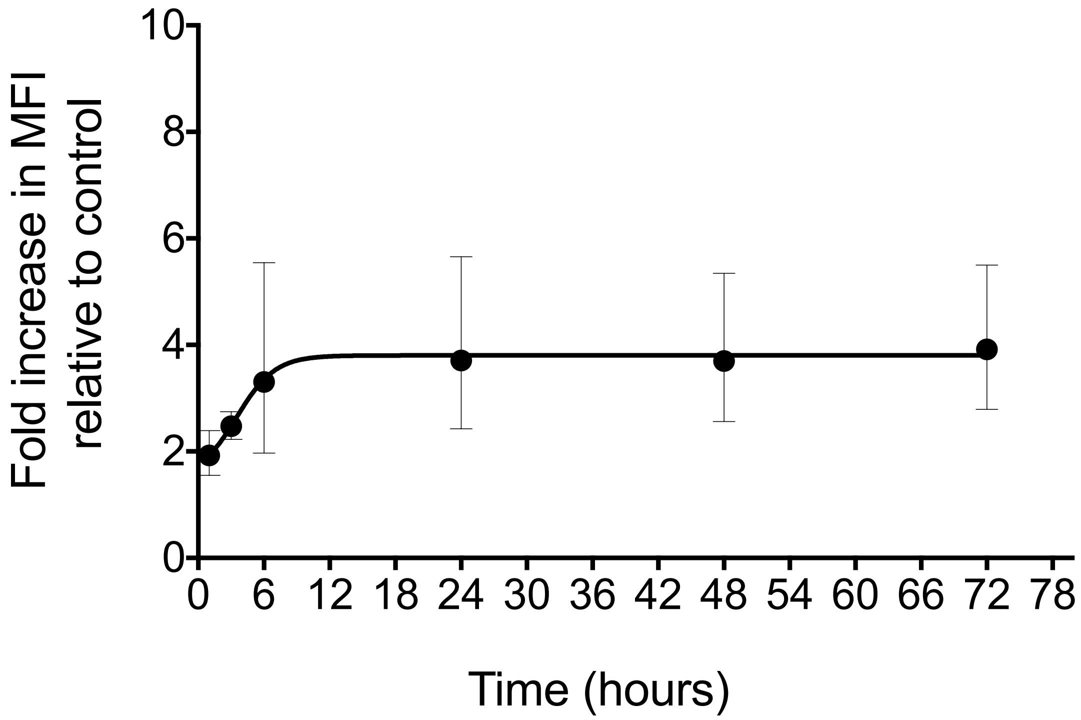

Figure S2. No further release of fluorescein is observed following prolonged incubation with fluorescein-loaded PMs. Human bone marrow mononuclear cells were incubated with a concentration of 5 x 10^{12} fluorescein-loaded PMs/ml for either1, 3, 6, 24, 48 or 72 hours and then analysed by flowcytometry. Data presented as mean ± S.D of three independent patients.

Supplementary figure 3

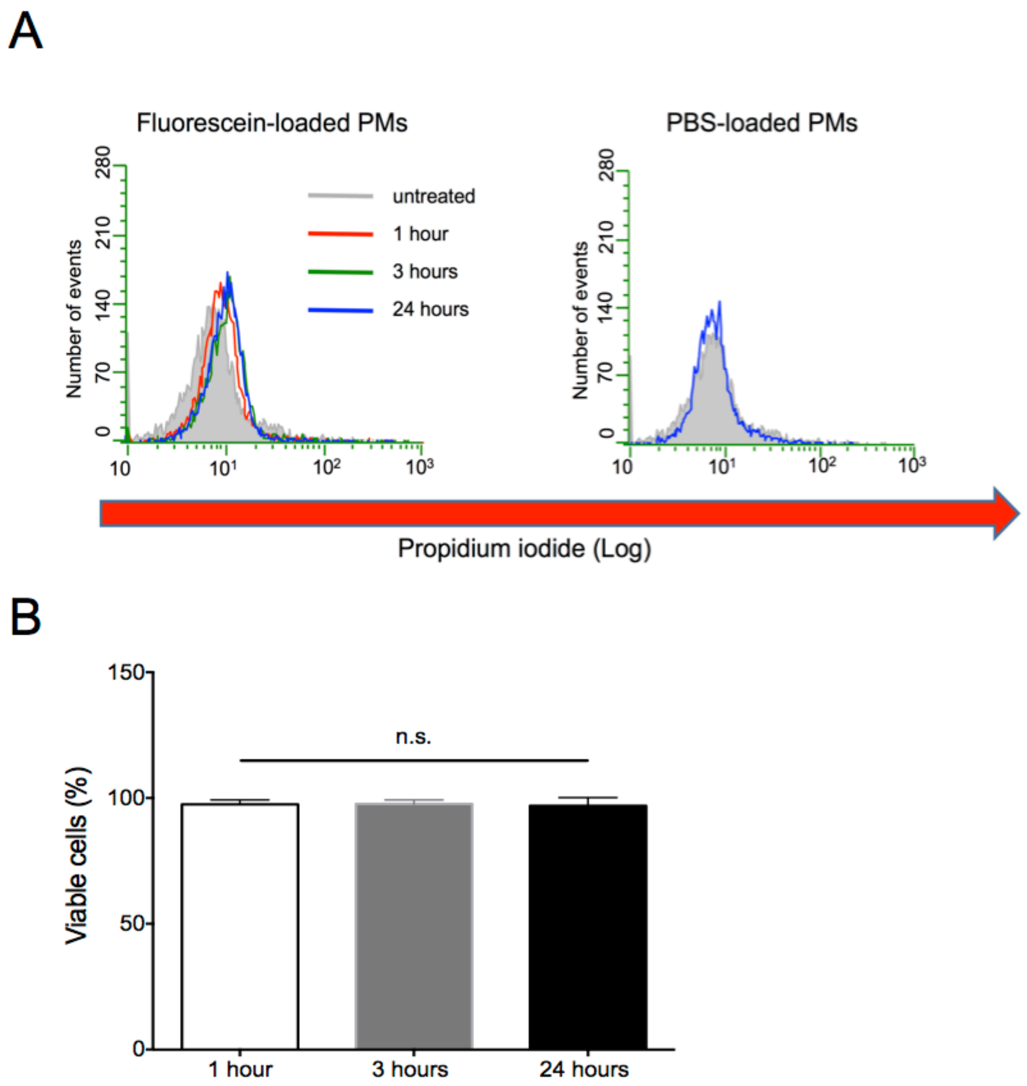

Figure S3. Loaded PMs do not induce cytotoxicity. (A) Flow cytometry of L929 cells incubated for 1 (red), 3 (green) or 24 hours (blue) with either fluorescein-loaded PMs or PBS-loaded PMs and stained with propidium iodide (1 mg/ml). (D) Percentage of viable cells after 1, 3 or 24 hours incubation with fluorescein-loaded PMs. Data presented as mean ± S.D. Statistical analysis is Kruskal-Wallis One-Way ANOVA on ranks. ns = non significant.

Supplementary Figure 4

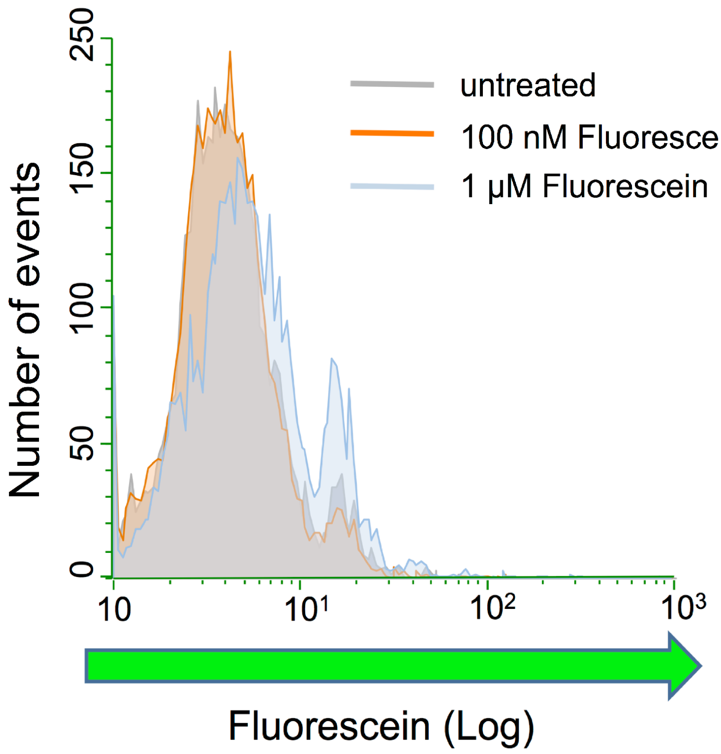

Figure S4 Equivalent concentrations of unencapsulated fluorescein induce negligible fluorescence. Flow cytometry of L929 cells incubated for 24 hours with either 100 nM (orange) or 1 μM (blue) fluorescein resulting in negligible fluorescence compared to untreated cells (grey).

Supplementary figure 5

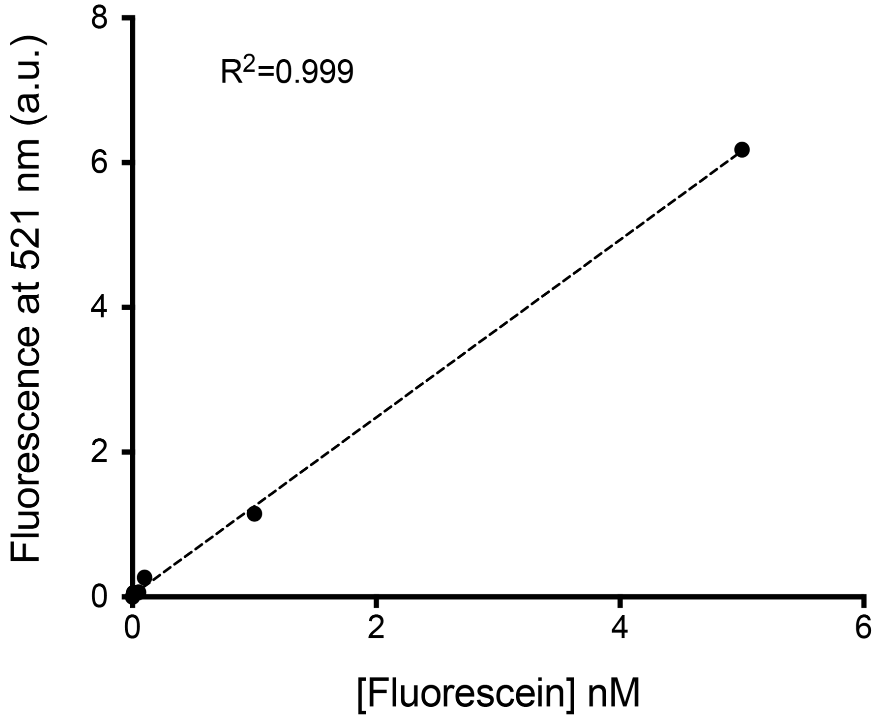

Figure S5. Standard curve of fluorescein fluorescence. Graph depicting the intensity of fluorescence of increasing concentrations of fluorescein in a solution containing cell lysate.

Supplementary figure 6

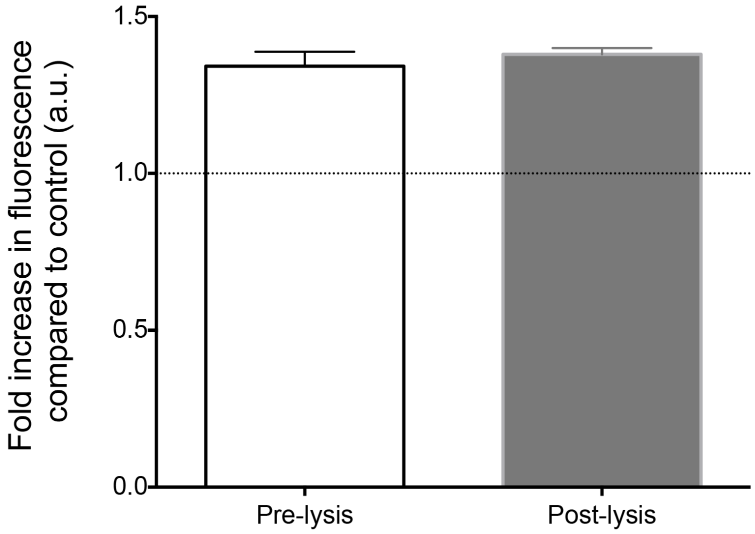

Figure S6. Fluorescence pre- and post-lysis. Relative levels of fluorescence of L929 cells incubated with fluorescein-loaded PMs pre- and post-lysis. Fluorescence is normalized against autofluorescence from control cells where no fluorescein-loaded PMs were added (dashed line). Data presented as mean ± S.D.
